# Getting Married

**Published:** 1866-12

**Authors:** 


GETTING MARRIED.

Into this gulf of perdition will they arrive at no remote day who arrange to go to boarding as soon as married, instead of going to housekeeping. A more unprofitable mistake cannot possibly be made by the newly married ; it is more mischievious in winter than in summer, for in warm weather the young wife can walk on the street, or drive in the Park, or visit among her friends, but in the winter they are cooped up in one room, and spend hours at the window-pane, gazing listlessly out opon the street ; afraid of the cold, yet detesting the confinement, while a great part of the time, the husband being at his business, the mind of the young wife is the prey of a thousand disquietudes ; at one time she thinks her friends are slighting her ; at another, she becomes envious of others who seem to be able to dress more elegantly than herself, and she begins to make unfavorable comparisons to herself and to her husband, as to their worldly condition, and she becomes moody, or petulant, and complains, and the husband wakes up to a new discovery, and as unwelcome as it is new—that his wife is not happy ; not happy in him, not happy in herself, not happy in her social position. But suppose these same persons had begun life in the old fashioned way ; had taken a small house and had busied themselves first in arranging whatever parental affection had bestowed, and then had set about deciding what they could afford to take from their own store of money and expend it for the most necessary articles of furniture, and as to the things they wanted besides, things not indispensable, yet desirable, laying plans together how, by economizing, first in one direction and then in another, they might lay aside enough to procure what they had set their minds upon ; and when this was accomplished, then to have another aim. No man can doubt that in going to housekeeping in such a manner, the prospect of a happy and thrifty married life is unmistakably more promising than in the health and heart destroying practice of living the first twelve months of married life in hotels and boarding houses. Very few young wives are safe in any public associations. The papers abound in cases of infidelity to marital engagements by lately marri ed girls being thrown into the society of men of leisure, their husbands being engaged in their business pursuits. The mere dandy, loafer, or gentleman of leisure has everv opportunity of taste, and dress, and address above that of the mere man of business, to turn away a woman's heart from her husband by the mere fact of causing her at first, without implicating himself, to draw unfavorable comparisons against her husband, or to personal tidiness. One of the most splendid weddings that had ever taken place then, in New York, resulted in a seperation, and a broken heart, and a premature grave within a few years, by the wife's continual twitting her husband, when they were walking on Broadway or the Avenue, about his want of taste in the selection of his apparel; —"why don't you bow as Mr. Blank does?"—"His gloves are in perfect taste; yours are such as a countryman would select."

These things grew upon her, while they alienated him, and living as they did at the finest hotel in the city, with uncounted gold at their command, and nothing to compel their attention away from trifling things, their minds dwelt upon them, magnified them, allowed them to see nothing else, with the result of a seperation under circumstances which were infinitely worse than death, for then the grave would have closed over every sorrow, whereas life was spared, with its long years of agony.

